# Identification of Dehydroxytrichostatin A as a Novel Up-Regulator of the ATP-Binding Cassette Transporter A1 (ABCA1)

**DOI:** 10.3390/molecules16097183

**Published:** 2011-08-25

**Authors:** Yang Xu, Yanni Xu, Yi Bao, Bin Hong, Shuyi Si

**Affiliations:** Institute of Medicinal Biotechnology, Peking Union Medical College and Chinese Academy of Medical Sciences, Beijing 100050, China

**Keywords:** dehydroxytrichostatin A, atherosclerosis, ABCA1, up-regulator, HDAC inhibitor

## Abstract

The ATP-binding cassette transporter A1 (ABCA1) mediates the cellular efflux of excess cholesterol and phospholipids to lipid-poor apolipoprotein A-I (apoA-I). ABCA1 plays an important role in high-density lipoprotein (HDL) biogenesis and reverse cholesterol transport. By using a cell-based screening model for the ABCA1 up-regulator and column chromatography, an active compound, 9179B, was isolated. Through analysis of its NMR data, 9179B was identified as dehydroxytrichostatin A. We found that 9179B increased the transcription of ABCA1 in a cell-based reporter assay, with an EC_50_ value of 2.65 μM. 9179B up-regulated ABCA1 expression at both mRNA and protein levels in HepG2 and RAW264.7 cells. It also up-regulated the expression of scavenger receptor class B type I (SR-BI) as well as the uptake of DiI-HDL in RAW264.7 cells. This compound stimulated ApoA-I-mediated cellular cholesterol efflux from RAW 264.7 cells. We further found that 9179B was a potent histone deacetylase (HDAC) inhibitor with an IC_50_ value of 0.08 μM. Reporter gene assays showed that the regulation of ABCA1 transcription by 9179B was mainly mediated by the −171/−75 bp promoter region. Together, our results indicate that 9179B is an ABCA1 up-regulator and dehydroxytrichostatin A may be a novel anti-atherogenic compound.

## 1. Introduction

Cardiovascular disease (CVD) is a major threat to human health and atherosclerosis (AS) is the principal pathogenesis of CVD. AS is a chronic inflammatory disease that is triggered by the build-up of cholesterol-rich plaques in the intima of arteries [1]. Consistently, epidemiological studies have shown a strong inverse relationship between plasma high-density lipoprotein cholesterol (HDL-C) levels and the incidences of CVD [2,3]. Other studies demonstrate that low total cholesterol and high HDL-C levels are associated with low morbidity and mortality rates of CVD [4]. Considerable evidence shows that therapeutically elevating HDL-C levels may be beneficial for CVD.

The ATP-binding cassette transporter A1 (ABCA1) is a membrane transporter, which is highly expressed in placenta, brain, adrenal glands and liver [5]. It mediates the rate-controlling step in HDL particle formation as well as the efflux of cellular phospholipids and cholesterol to lipid-free apolipoprotein A-I (apoA-I), which may account for the atheroprotective effect of HDL [6,7]. The high basal expression of ABCA1 in hepatocytes facilitates the synthesis of pre-beta-HDL and promotes the efflux of cellular phospholipids and cholesterol to lipid-free apoA-I to form nascent HDL [8]. Furthermore, ABCA1 in the liver and intestine contributes to 80% and 20% of the HDL biogenesis, respectively [9]. Overexpression of hepatic ABCA1 raises HDL-C levels considerably [15,16,17]. Mutations of the ABCA1 gene can result in Tangier disease, a severe HDL deficiency characterized by the accumulation of cholesterol in tissues enriched in macrophages [10,11]. Increased ABCA1 expression in human macrophages prevents arterial inflammation [12]. The importance of ABCA1 in HDL metabolism and its atheroprotective effects have been confirmed in apoE knockout mice and in C57BL/6 mice with diet-induced AS [13,14]. Thus, ABCA1 could be a promising therapeutic target for CVD.

In our previous study, a high-throughput screening (HTS) method for ABCA1 up-regulators has been developed [18]. In the present study, 2,600 crude microbial secondary metabolite extracts were screened. Strain IA04-9179 is a positive hit in the HTS assay and the extract from this strain displays a potent activity in upregulating the transcription of ABCA1. In this report, we identify 9179B as the active compound produced by strain IA04-9179. The chemical structure of 9179B was identified as dehydroxytrichostatin A by NMR. Dehydroxytrichostatin A inhibits histone deacetylase (HDAC) and increases ABCA1 expression in HepG2 and RAW 264.7 cells. Furthermore, treatment of HepG2 cells with 9179B and the *cis*-elements in ABCA1 promoter up-regulates ABCA1 expression.

## 2. Results and Discussion

### 2.1. Isolation and Identification of 9179B from Strain 04-9179

**Figure 1:**
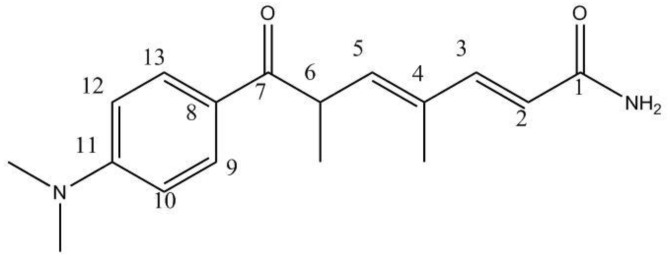
The chemical structure of 9179B.

Strain 04-9179 increased the luciferase activity of ABCA1p-LUC HepG2 cells. After the purification through column chromatography with HP20 macroporous resin, ODS reverse-phase column, and semipreparative HPLC, an active compound named 9179B was isolated. The UV spectrum of 9179B in methanol showed a λ_max_ of 266 and 350 nm. The purified compound was identified by ESI, FAB, ^1^H-NMR, ^13^C-NMR, and 2D NMR. The molecular formula of 9179B was established to be C_17_H_22_N_2_O_2_ by MS and NMR. The structure of 9179B is similar to trichostatin A by ^13^C-NMR (Table 1). The chemical shift of the olefin carbon (C-2) was decreased in 9179B, which suggests a different substituent group at the C-1 carbonyl carbon. The ESI and HMBC results showed that for 9179B, a hydrogen atom is substituted for the hydroxyl group in the hydroxamic acid of trichostatin A. Therefore, the structure of 9179B was identified as the trichostatin derivative 7-(4-(dimethylamino)phenyl)-4,6-dimethyl-7-oxohepta-2,4-dienamide (Figure 1) or dehydroxytrichostatin A.

**Table 1:** ^1^H-NMR and ^13^C-NMR data of the compound, 9179B.

Position	^13^C-NMR ^a^	^1^H-NMR ^b^
1	n.d. ^c^	
2	120.1	5.97 (1H, d, *J* = 15.5)
3	146.9	7.10 (1H, d, *J* = 15.5)
4	134.3	
5	141.1	5.86 (1H, d, *J* = 9.5)
6	41.6	4.48 (1H, m)
7	201.4	
8	124.6	
9,13	131.8	7.81 (2H, d, *J* = 9.0)
10,12	111.9	6.67 (2H,d, *J* = 9.0)
11	155.4	
4-Me	12.7	1.80 (3H, s)
6-Me	18.2	1.22 (3H, d, *J* = 6.5)
N-Me_2_	40.2	3.02 (6H, s)

^a^: 500MHz in CD_3_OD; ^b^: 500MHz in CD_3_OD; ^c^: Not detected.

### 2.2. Effect of 9179B and Other HDAC Inhibitors on ABCA1 Promoter Activity

**Figure 2:**
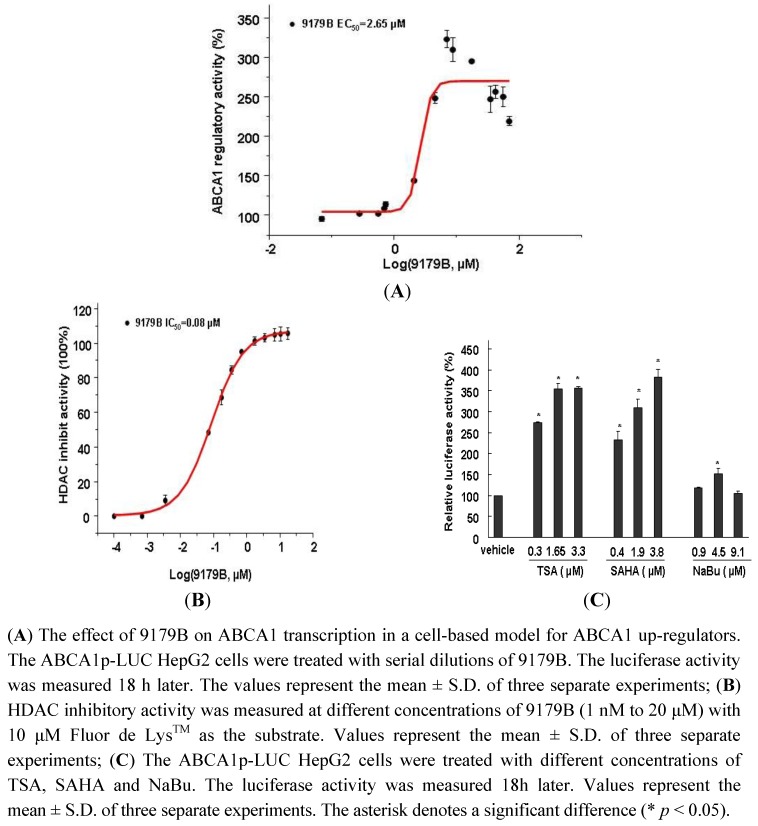
The effect of 9179B and other HDAC inhibitors on ABCA1 promoter activity.

To determine the bioactivity of 9179B on the transcriptional activation of the ABCA1 gene promoter, a dose-response curve of luciferase activity was determined in ABCA1p-LUC HepG2 cells. 9179B increased the luciferase activity in ABCA1p-LUC HepG2 cells in a dose-dependent manner, with an EC_50_ value of 2.65 μM. 9179B strongly induced the transcription driven by the ABCA1 promoter, reaching more than 3-fold at 6.99 μM (Figure 2A).

9179B is a derivative of trichostatin A (TSA), an inhibitor of histone deacetylase (HDAC) [19,20,21]. To test the inhibitory effect of 9179B on HDAC, the enzyme activities in the presence of different concentrations of 9179B were measured. Figure 2B shows that 9179B inhibits HDAC activity in a dose-dependent manner, with an IC_50_ value of 0.08 μM. These results indicate that 9179B is a potent HDAC inhibitor.

To investigate whether the up-regulation of ABCA1 promoter activity by 9179B is due to its inhibition of HDAC, three other HDAC inhibitors, TSA, suberoylanilide hydroxamic acid (SAHA) and sodium butyrate (NaBu), were used to compare their inhibition of ABCA1 transcription in ABCA1p-LUC HepG2 cells. These three inhibitors also increased the transcriptional activities of ABCA1 promoter, and reached at a maximum value of 355% at 3.3 μM, 381% at 3.8 μM and 152% at 4.5 μM, respectively (Figure 2C).

The transcriptional regulation of ABCA1 is a highly complex process. Nuclear receptors involved in lipid homeostasis require the recruitment of coactivators and corepressors, collectively termed coregulators [22]. Coactivators are capable of recruiting the basal transcription factors and histone acetylases (HAT), which open the chromatin structure and induce transcription. Corepressors inhibit transcription *via* HDACs [23]. For example, the nuclear receptor corepressor (N-CoR), the silencing mediator of retinoic acid, and thyroid hormone receptors (SMRT) are essential components of the corepressor complex and they repress transcription by activating HDACs in the complex [24,25,26]. Therefore, this is the first report showing that 9179B is a HDAC inhibitor (HDACi) (Figure 2B) and can remarkably increase ABCA1 expression as well. We speculate that 9179B promotes ABCA1 transcription by modulating histone deacetylation in the promoter region of ABCA1 gene.

A HDAC inhibitor TSA was noticed to decrease the expression of SRA in P388D1 macrophage, suggesting that HDAC inhibition may prevent the development of AS [27]. Inflammation contributes to the formation and progression of AS and HDAC inhibitors have been shown to exhibit anti-inflammatory activities. Anti-inflammatory treatment regimens used in tumor necrosis factor-α blockage, IL-1 receptor antagonism, and leukotriene blockage may be beneficial for patients with coronary artery disease. Treatment of inflammation in atherosclerotic cardiovascular disease is thought to be emerging therapies [28,29]. 

### 2.3. The Effects of 9179B on ABCA1 Expression in HepG2 and RAW 264.7 Cells and on Cholesterol Efflux in RAW 264.7 Cells

**Figure 3:**
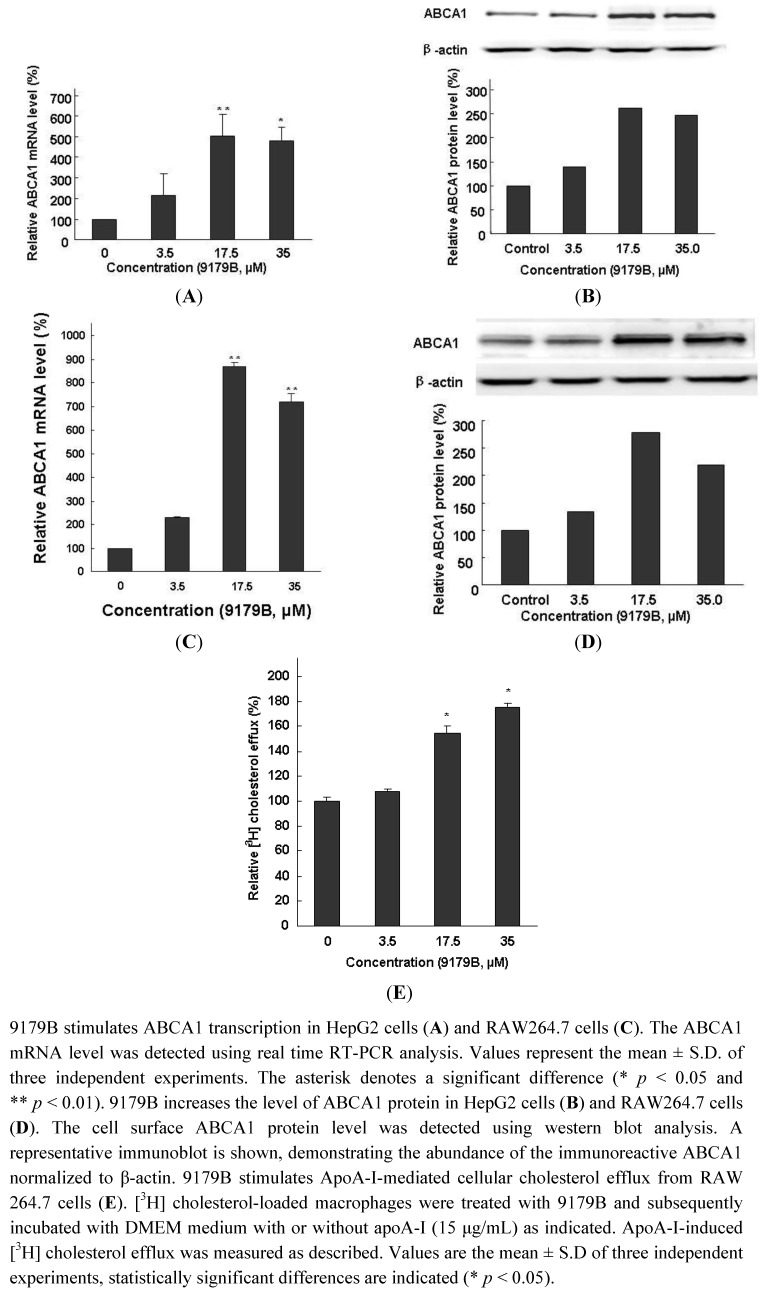
The effects of 9179B on the expression of ABCA1 in HepG2 and RAW 264.7 cells andon cholesterol efflux in RAW 264.7 Cells.

To determine whether the induction of ABCA1 expression by 9179B was due to the up-regulation of transcription, quantitative real time RT-PCR was performed to investigate the abundance of ABCA1 mRNA and protein in HepG2 cells with and without 9179B treatment. Our data showed that 9179B increased the mRNA expression of ABCA1 in a dose-dependent manner in HepG2 cells, reaching about 5 folds at 17.5 μM (Figure 3A

In agreement, the protein expression of ABCA1 was also increased by 9179B treatment in a dose-dependent manner in HepG2 cells, reaching about 1.6-fold at 17.5 μM (Figure 3B). Cholesterol may enter macrophages *via* several different pathways and induce the transformation of macrophages into foam cells, the first step in atherosclerotic lesion development. ABCA1-dependent cholesterol efflux to lipid-poor apoA-I is a crucial factor in the prevention of excessive cholesterol accumulation in macrophages of the arterial wall and their transformation into foam cells [1]. Therefore, the effect of 9179B on ABCA1 expression in RAW264.7 cells was examined. Quantitative real time RT-PCR analysis indicated that 9179B increased the mRNA expression of ABCA1 in a dose-dependent manner in RAW 264.7 cells, reaching more than 8-fold higher at 17.5 μM (Figure 3C Consistently, the protein expression of ABCA1 was increased by 9179B treatment in a dose-dependent manner in RAW264.7 cells, reaching about 1.8-fold at 17.5 μM (Figure 3D).

Taken together, our data clearly demonstrate that the small molecule 9179B increases ABCA1 expression at both mRNA and protein levels in HepG2 and RAW 264.7 cells. Previous studies have suggested that ABCA1 is anti-atherogenic. Hepatocyte and macrophage ABCA1 plays important roles in HDL biosynthesis and cellular cholesterol efflux, respectively [4,30]. It is thus conceivable that 9179B as a novel and potent ABCA1 up-regulator may be beneficial for the prevention of atherosclerotic diseases.

In order to investigate the potential role of the active compound 9179B in modulating cholesterol efflux from mouse peritoneal macrophages, [^3^H] cholesterol efflux assay was performed using RAW264.7 cells. As shown in Figure 3E, treatment of RAW264.7 cells with 9179B resulted in a significant increase in cholesterol efflux to extracellular ApoA-I. The ratio of [^3^H] cholesterol efflux was 154.5% and 175.6% at 17.5 μM and 35 μM of 9179B, respectively.

Removal of cholesterol from macrophages *via* RCT is of significant importance in the prevention of atherosclerosis development. ABCA1 plays a pivotal role in RCT, and facilitates the efflux of cellular cholesterol to ApoA-I [31,32]. Drugs that induce ABCA1 expression in mice increased clearance of cholesterol from tissues [33]. 9179B could upregulate ABCA1 expression in HepG2 and RAW264.7 cells. It could be concluded that [^3^H] cholesterol efflux induced by 9179B was the result of the upregulation of ABCA1 expression. This is a benefit to the prevention of atherosclerosis.

### 2.4. The Influence of 9179B on SR-BI and the Uptake of DiI-HDL in RAW 264.7 Cells

**Figure 4:**
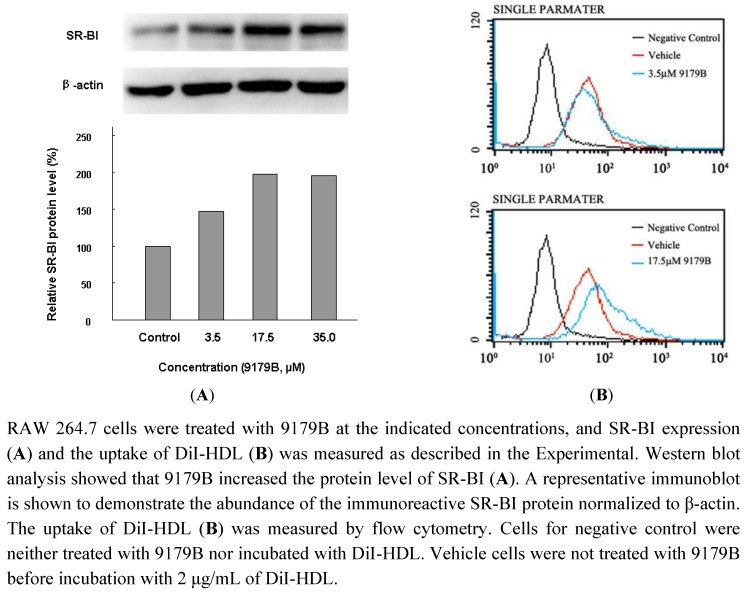
The effect of 9179B on the expression of SR-BI in RAW 264.7 cells and flow cytometry analysis of the uptake of DiI-HDL by RAW 264.7 cells.

IA04-9179 was also found to upregulate CLA-1 (human homologue of SR-BI) in a CLA-1-LUC cell line. In agreement with this finding, as a known compound trichostatin A (TSA), 9179A has been isolated and shown to upregulate CLA-1/SR-BI expression [34]. Similar effects on the expression of SR-BI were observed for 9179B. RAW264.7 cells were used to examine the effect of 9179B on SR-BI. After treatment with 3.5 μM, 17.5 μM and 35.0 μM 9179B, Western blot assay showed that 9179B could up-regulate SR-BI expression in a dose-dependent manner (Figure 4A). At 35.0 μM 9179B could significantly increase the SR-BI protein levels by 97.1%.

SR-BI is the main receptor of HDL and it is expressed in macrophages and plays an important role in the process of reverse cholesterol transport (RCT) [21]. Since 9179B increases the expression level of SR-BI, we tested whether 9179B could enhance the uptake of HDL. The uptake of fluorescently labeled DiI-HDL was measured after 4 h incubation in the presence of different concentrations of 9179B or vehicle (0.1% DMSO) in RAW 264.7 cells. The results showed that 3.5 μM and 17.5 μM 9179B promotes DiI-HDL uptake into RAW 264.7 cells by 29.5% and 153%, respectively (Figure 4B).

9179B increases the uptake of DiI-HDL in RAW264.7 cells, which may be caused by the up-regulation of SR-BI expression by 9179B. The increased uptake of DiI-HDL might result in cholesterol efflux from macrophages. Taken together, our results suggest that 9179B may facilitate the efflux of cellular cholesterol to extracellular acceptors through the induction of ABCA1 and SR-BI expression and/or their activities, which will benefit to AS.

### 2.5. The Influence of 9179B on Different Cis-Regulatory Elements of ABCA1

**Figure 5:**
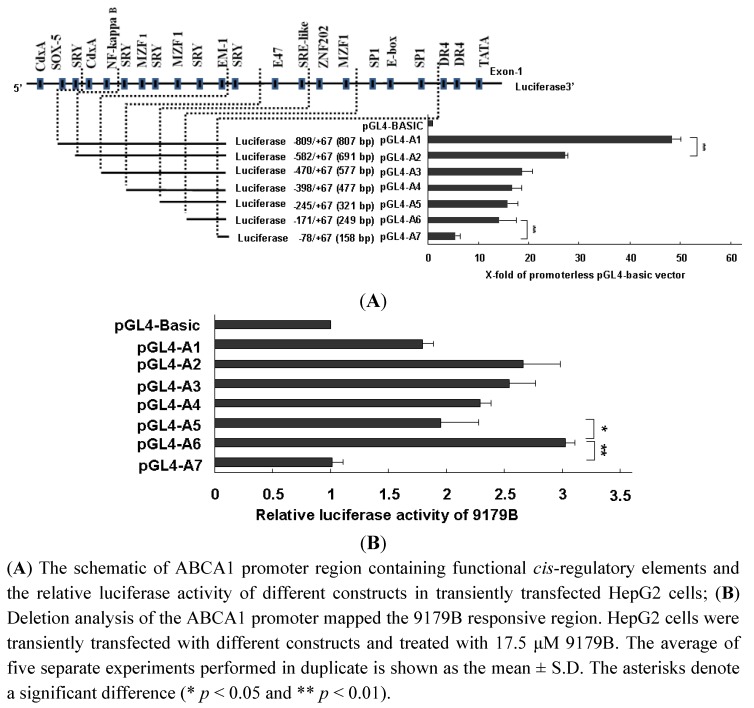
Characterization of the DNA motifs mediating 9179B up-regulation of ABCA1 promoter activity.

To identify the putative response domain in the ABCA1 promoter, seven different promoter-fragment/luciferase reporter constructs with progressive 5’ end deletions were transiently expressed in HepG2 cells. The basal promoter activity decreased significantly upon removal of the base pairs between −719 and −582. Removal of the base pair from −171 to −78 further reduced the promoter activity (Figure 5A). We tested whether 9179B could induce transcription with these fragments of ABCA1 promoter. Figure 5B shows that the transcriptional activity between −171/+67 bp and −78/+67 bp fragments was significantly different. 

When the −171/−78 bp fragment was removed, the luciferase activity decreased dramatically. This indicates that the −171/−78 bp fragment is important for 9179B to induce ABCA1 transcription. In this study, the *cis*-element of the ABCA1 promoter responsible for the 9179B up-regulation was mapped to the −171/−78 bp fragment (Figure 5B). Previous studies showed that two SP1 sites (the −91 and −157 GnC motifs) and an E-box (−140) motif were identified within the −171/−75 bp region [35,36]. SP1 plays a critical role in the formation or recruitment of the transcription initiation complex at the core promoter and also regulates the expression of proteins and enzymes involved in lipoprotein metabolism [35,36]. Overexpression of SP1 increased ABCA1 mRNA expression in HeLa cells. The E-box motif mediates transcriptional repression of the ABCA1 gene *via* basic helix-loop-helix transcription factors [35,37]. Removing the −171/−78 bp fragment, the luciferase activity decreased significantly. Therefore, we reason that the *cis*-element SP1 contributes to the up-regulation of the ABCA1 transcription by 9179B. These results suggest that 9179B may activate ABCA1 gene transcription by inhibiting deacetylase activity, or by activating the SP1 *cis*-element or through both mechanisms.

## 3. Experimental

### 3.1. General

The HDAC Fluorimetric Assay/Drug Discovery Kit was obtained from Enzo Life Science (Plymouth Meeting, PA, USA). The rabbit polyclonal antibody for ABCA1 was purchased from Novus Biologicals (Littleton, CO, USA). 1,1'-Dioctadecyl-3,3,3',3'-tetramethylindocarbocyanine perchlorate-labeled HDL (DiI-HDL) was purchased from Biomedical Technologies (Stoughton, MA, USA). White-wall, clear bottom 96-well polystyrene assay plates were obtained from BMG LABTECH (Offenburg, Germany). 60-mm dishes and 24- and 96-well plates for cell culture were obtained from Corning (Acton, MA, USA). Cell culture media and fetal bovine serum (FBS) were bought from Hyclone (Logan, UT, USA). G418 was obtained from Invitrogen (Carlsbad, CA, USA). 

### 3.2. Cell Culture

HepG2 human hepatoma cells were grown in minimum essential medium with Earle’s balanced salts and 2 mM L-glutamine solution (MEM-EBSS) containing 10% FBS (medium A). ABCA1p-LUC HepG2 cells, which are HepG2 cells stably transfected with a gene construct in which the promoter and regulatory control elements for the human ABCA1 gene were fused to a luciferase reporter gene. The transfected cells were grown in medium A supplemented with 600 μg/mL G418 (medium B). RAW264.7 murine macrophage cells were grown in Dulbecco’s modified Eagle’s medium (DMEM, high glucose) containing 10% FBS (medium C). All cells were maintained in a humidified 5% CO_2_ incubator at 37 °C. 

### 3.3. The Isolation, Purification and Structure Elucidation of 9179B

The fermentation of strain 04-9179 was performed as previously described [31]. The fermentation filtrate (4 × 30 liters) was applied to a HP-20 macroporous resin column (Mitsubishi Chemical, Tokyo, Japan), which was washed with water and eluted with aqueous acetone (30%, 50%, 80%, 100%). The 50% acetone eluting fractions with enhanced luciferase activity were collected and concentrated *in vacuo*. The dry residue was dissolved in methanol and loaded on a 4 × 300 cm column of octadecyltrichlorosilane (ODS; YMC, Kyoto, Japan). The column was eluted with a three-column bed volume of aqueous methanol (10%, 20%, 30%, 40%, 45%, 50%, 55%, 60%, 80%, 100%). The eluates were analyzed by high performance liquid chromatography (ODS column, 150 × 4.6 mm, 5 μm, Shimadzu, Kyoto, Japan) with MeOH-water (50:50) as eluent. The fractions that can upregulate ABCA1 transcription were evaporated and concentrated *in vacuo. *Final purification was achieved using semipreparative HPLC (250 × 9.4 mm, 5 μm, Agilent, CA, USA), which was eluted with 52% MeOH-water. The pure active compound, 9179B, was obtained and concentrated to dryness. The physicochemical properties of 9179B were determined *via* UV spectrometry (UV-200S, Shimadzu, Kyoto, Japan), mass spectrometry (JMS-DX 300, Jeol, Tokyo, Japan), and NMR spectrometry (XL-400, Varian, Palo Alto, CA, USA). The structure of 9179B was elucidated by spectral data from UV, ESI, FAB, ^1^H-NMR, ^13^C-NMR, HSQC, HMBC, and DEPT with CD_3_OD as the solvents.

### 3.4. ABCA1 Promoter Activity Assay

The ABCA1 transcriptional activity assay was carried our as described previously [18]. Briefly, the ABCA1p-LUC HepG2 cells were plated in 96-well dishes at 500,000 cells/well in 100 μL medium B for 12 h. Then the cells were treated with the indicated concentrations of 9179B or vehicle (0.1% DMSO). After being incubated for 18 h at 37 °C, cells were washed with PBS (137 mM NaCl, 2.7 mM KCl, 4.3 mM Na_2_HPO_4_, 1.4 mM KH_2_PO_4_, pH 7.3), and then the luciferase activity was detected as relative luminescence units (RLUs) using the Luciferase Assay System (Promega, Madison, WI, USA).

### 3.5. Enzyme Inhibitor Activity Assay and Other HDAC Inhibitor on ABCA1 Promoter Activity

HDAC inhibition activity was assayed using the Fluorimetric Assay/Drug Discovery Kit (BML-AK500) according to the manufacturer’s protocol. The fluorescence intensity was measured using a fluorescence microplate reader (EnVision Multilabel Reader, PerkinElmer, Waltham, MA, USA) (excitation 360 nm, emission 460 nm). The ability of 9179B to inhibit HDAC activity was measured at the indicated concentrations of 9179B with 10 μM Fluor de Lys as substrate. The IC_50_ was calculated using Origin 7.5 software. The ABCA1 transcriptional activity assay was performed as described in 2.2. The ABCA1p-LUC HepG2 cells were treated with the indicated concentrations of TSA, SAHA, NaBu or vehicle (0.1% DMSO). After incubation for 18 h at 37 °C, cells were washed with PBS, and then the luciferase activity was detected as relative luminescence units (RLUs) using the Luciferase Assay System (Promega, Madison, WI, USA).

### 3.6. The Effects of 9179B on ABCA1 Expression in HepG2 or RAW264.7 Cells

HepG2 or RAW264.7 cells were incubated with 9179B at the indicated concentrations for 24 h and then were washed twice with PBS. Total cellular RNA and protein extracts were obtained as described before [18]. 

For quantitative real time PCR, SYBR Green PCR Core Reagent (Applied Biosystems, Foster City, CA, USA) was used on an iQ5 Multicolor Real-Time PCR Detection System (Bio-Rad, Hercules, CA, USA). The following sequences of primers (sense and antisense) were used for amplification: 5’-GCCTGCTAGTGGTCATCCTG-3’ and 5’-CCACGCTGGGATCACTGTA-3’ (for human ABCA1), 5’-TCCACTGCGTCTTCACC-3’ and 5’-GGCAGAGATGATGACCCTTTT-3’ (for human GAPDH); 5’-GGGTCTGAACTGCCCTACCT-3’ and 5’-TACTCCCCTGATGCCACTTC-3’ (for mouse ABCA1), 5’-CTAAGGCCAACCGTGAAAG-3’ and 5’-ACCAGAGGCATACAGGGACA-3’ (for mouse β-actin). Expression data were normalized for GAPDH and β-actin levels.

For Western blotting, protein extracts (25 μg) from control or treated cells were subjected to 10% SDS-polyacrylamide gel electrophoresis. Then proteins were transferred to a 0.45-μm polyvinylidene difluoride (PVDF) membrane (Millipore, Bedford, MA, USA). The membrane was blocked for 1h at room temperature in TBS contain 0.1% Tween-20 (TBST) in the presence of 5% nonfat dry milk. Membranes were incubated with the rabbit polyclonal antibody for ABCA1 protein (1:500 dilution, 4 °C, overnight) (Novus Biologicals, Littleton, CO, USA) in TBST containing 5% nonfat dry milk. Then membranes were washed by TBST and incubated with horseradish peroxidase-conjugated goat antirabbit IgG (Novus Biologicals) in TBST containing 5% nonfat dry milk for 1h at room temperature (1:2000 dilution). An enhanced chemiluminiscence detection system (Santa Cruz Biotechnology, Santa Cruz, CA, USA) was used for detection. Human β-actin was simultaneously detected as an internal control.

### 3.7. The Effects of 9179B on Cholesterol Efflux in RAW 264.7 Cells

The cholesterol efflux assay was done as previously described [38]. RAW264.7 cells were plated in 96-well plates and incubated for 24 h. Then they were incubated with DMEM containing 2 μCi of [1,2-^3^H] cholesterol (1 mCi/mL; PerkinElmer, Waltham, MA,USA) per mL for 24 h. Then the cells were washed three times with PBS containing 0.1% BSA (PBS-BSA), and fresh DMEM containing 0.1% BSA (DMEM-BSA) with 9179B at the indicated concentrations added for 24 h. After intensive washing of cells with PBS-BSA, ApoA-I (15 μg/mL) prepared in DMEM-BSA as the cholesterol acceptor were added and incubated for 24 h. Finally, medium was collected and centrifuged (10,000 × g for 5 min), and the radioactivity was counted by liquid scintillation counting. The residual radioactivity in the cell fraction was determined after half hour hydrolysis with 0.1 M NaOH. The ratio of [^3^H] efflux was calculated by dividing the radioactive counts in the medium by the sum of the radioactive counts in the medium and the cell fraction. DMEM-BSA was used as the blank, and the efflux of vehicle treated cells was expressed as 100%.

### 3.8. The Effect of 9179B on SR-BI Expression in RAW264.7 Cells and the Analysis of Cellular Uptake of DiI-Labeled HDL by Flow Cytometry

RAW264.7 cells were incubated with 9179B at the indicated concentrations for 24 h and then were washed twice with PBS. For Western blots, protein extracts (25 μg) from control or treated cells were subjected to 10% SDS-polyacrylamide gel electrophoresis. Then proteins were transferred to a 0.45-μm polyvinylidene difluoride (PVDF) membrane (Millipore, Bedford, MA, USA). The membrane was blocked for 1h at room temperature in TBS contain 0.1% Tween-20 (TBST) in the presence of 5% nonfat dry milk. Membranes were incubated with the rabbit polyclonal antibody SR-BI (1:1,000 dilution, 4 °C, overnight) (Novus Biologicals, Littleton, CO, USA) in TBST containing 5% nonfat dry milk. Then membranes were washed by TBST and incubated with horseradish peroxidase-conjugated goat antirabbit IgG (Novus Biologicals) in TBST containing 5% nonfat dry milk for 1 h at room temperature (1:3000 dilution). An enhanced chemiluminiscence detection system (Santa Cruz Biotechnology, Santa Cruz, CA, USA) was used for detection. Mouse β-actin was simultaneously detected as an internal control to monitor the intensity of the immunoreactive SR-BI.

RAW264.7 cells were plated at 50,000 cells/well in 24-well dishes. The cells were treated for 18 h with 9179B at specified concentrations. Then DiI-HDL (final concentration 2.0 μg/mL) was added at 37 °C for 4 h. The cells were washed twice with cold PBS and incubated in PBS supplemented with 0.5% bovine serum albumin and 2 mM EDTA at 4 °C for 1 h. The cell suspensions were transferred and centrifuged (3 min, 800 g, 4 °C) and then were resuspended in PBS. DiI fluorescence was analyzed by an Epics XL flow cytometer (Coulter Corporation, Miami, FL, USA).

### 3.9. Luciferase Reporter Assay

The reporter constructs contained the ABCA1 upstream regulatory sequence region from −719 to −67 bp (nucleotide +1 corresponds to the A of the ATG start codon). pGL4-ABCA1-1 (−719/+67, 807 bp), pGL4-ABCA1-2 (−582/+67, 691 bp), pGL4-ABCA1-3 (−470/+67, 577 bp), pGL4-ABCA1-4 (−398/+67, 477 bp), pGL4-ABCA1-5 (−245/+67, 321 bp), pGL4-ABCA1-6 (−171/+67, 249 bp) and pGL4-ABCA1-7 (−78/+67, 158 bp) were purified using PureYield^TM^ Plasmid Midiprep System (Promega). The reporter plasmid (0.2 μg) was transfected into 50,000 HepG2 cells using the cationic liposome transfection method (Lipofectamine 2000, Invitrogen). After reporter plasmids were transfected for 5 h, then HepG2 cells were incubated with 17.5 μM 9179B or vehicle (0.1% DMSO) in MEM medium without FBS. Cells were incubated for 18h and the luciferase activity was detected as RLUs by the Luciferase Assay System (Promega).

### 3.10. Statistical Analysis

All experimental results were expressed as means ± S.D. Statistical significance of the data was evaluated by the Student’s *t*-test. A significant difference was indicated at *p * < 0.05.

## 4. Conclusions

In this paper we report for the first time the identification and characterization of dehydroxytrichostatin A (9179B) as a novel up-regulator of ABCA1. Dehydroxytrichostatin A upregulates ABCA1 expression at the mRNA and protein levels in HepG2 cells and RAW264.7 cells, and increases DiI-HDL uptake in RAW264.7 cells. 9179B stimulated ApoA-I-mediated cellular cholesterol efflux from RAW 264.7 cells. Moreover, we found that 9179B was a potent HDAC inhibitor. Our results also suggest that 9179B may regulate ABCA1 transcription by affecting the interaction of HDAC or SP1 with the ABCA1 promoter. These results show the significance of further studies of ABCA1 transcriptional regulation. In conclusion, our studies have demonstrated that 9179B is a novel up-regulator of ABCA1, which could be used as an anti-atherogenic drug.
